# Insights into the Heat Shock Protein 70 (Hsp70) Family in *Camelina sativa* and Its Roles in Response to Salt Stress

**DOI:** 10.3390/plants13233410

**Published:** 2024-12-04

**Authors:** Parviz Heidari, Sadra Rezaee, Hadiseh Sadat Hosseini Pouya, Freddy Mora-Poblete

**Affiliations:** 1 Faculty of Agriculture, Shahrood University of Technology, Shahrood 3619995161, Iran; rezaee6265sadra@gmail.com; 2 Faculty of Science, Ferdowsi University of Mashhad, Mashhad 9177948944, Iran; hosseinipouya.h@gmail.com; 3 Institute of Biological Sciences, University of Talca, 1 Poniente 1141, Talca 3460000, Chile

**Keywords:** Hsp70 protein, co-expression interaction, salinity stress

## Abstract

Hsp70s, a group of heat shock proteins, are ancient proteins that play a crucial part in maintaining the stability of cells when faced with various internal and external stresses. In this research, there are 72 *CsHSP70* genes present and verified in *Camelina sativa*, all of which exhibit a wide range of physicochemical characteristics. Through evolutionary analysis, the Hsp70 family was categorized into five primary groups, and numerous segmental duplications were anticipated among the *CsHSP70* genes. The GO enrichment analysis of co-expression network elements revealed a significant association between key signaling terms, such as phosphorelay signal transduction, and MAPK cascade with the function of *CsHsp70*. An analysis of transcriptome data exposed to cold, drought, salinity, and cadmium stress demonstrated the varied expression profiles of *CsHsp70* genes. The expression levels of *CsHSP70* genes varied across various organs and stages of development in camelina, although some of them illustrated tissue-specific expression. qRT-PCR analysis further disclosed that *CsHsp70-60*, -*52*, and -*13* were up-regulated and *CsHsp70-03*, -*58*, and -*09* showed down-regulation in response to salinity. Furthermore, *CsHsp70* genes are categorized as late-responsive elements to salinity stress. Through docking analysis, the current research revealed that CsHsp70 proteins interacted with ABA, BR, and MeJA.

## 1. Introduction

Heat shock proteins (HSPs) are a crucial part of the plant stress response, as they help safeguard cell structures and keep a stable environment under tough conditions [1]. These proteins can be found in many organisms, from prokaryotes to eukaryotes [2]. HSPs serve as built-in biomarkers that are very important in genetic diversity and aiding survival of organisms exposed to environmental pressures [3,4,5]. In plant cells, HSPs protect cells by influencing how new proteins form and move, plus reforming damaged proteins both when under stress and not stressed [6,7]. These proteins also have major roles in cell signaling, which eventually helps control how certain genes express during stressful conditions [3,8,9]. The HSP family covers various kinds of proteins ranked according to their molecular weights and sequence matches. These include small HSP, chaperonin (Hsp60), Hsp70, Hsp90, and Hsp100, each with specific functions in the stress response. The weights of these proteins may range from 10 kDa to over 100 kDa [10,11,12,13,14]. Among these classes of protein is the preserved evolutionary protein called Hsp70. It plays a vital role in defending cellular homeostasis against various physiological challenges [15,16]. The structure of an Hsp70 protein consists of three unique domains: an N-terminal domain responsible for ATP hydrolysis, a substrate-binding domain (SBD), and a C-terminal domain [17,18,19].

In particular, plant Hsp70 proteins are distributed across various parts of the cell as the cytosol nucleus, endoplasmic reticulum, chloroplasts, mitochondria and plasma membrane [20,21,22]. The location inside the cell where a particular Hsp70 protein is found is determined by the regulatory motif of that protein present at its N-terminal and C-terminal ends [6,23,24,25]. Hsp70s undergo substantial post-translational modifications. The Plant PTM viewer records nearly 300 Hsp70 peptides, each exhibiting discernible post-translational modifications [26]. The most prevalent alterations include phosphorylation (observed in 65 peptides), acetylation (found in 53 peptides), sulfenylation (present in 33 peptides), and ubiquitination (identified in 27 peptides) [27]. Furthermore, it was stated that heat shock factors (Hsfs) control the expression of *HSP* genes. In terms of plants, HsfA, a category of Hsf, is known to improve plant resilience to stresses such as anoxia, heat, osmotic stress, and oxidative stress [28]. Research shows that Hsp70 affects how proteins in the abscisic acid (ABA) signaling pathway work during abiotic stress. It engages with and promotes the breakdown of ABA receptor proteins, thus controlling ABA signaling and the plant’s response to stress [29]. Hsp70s prevent oxidative damage by attaching to antioxidant enzymes that eliminate reactive oxygen species (ROS) [30].

The function and response of *HSP70* genes under stress conditions in plants have been studied. It was stated that the suppression of HSP70 expression in response to salinity stress affected various physiological parameters in plants, for instance, growth inhibition, alterations in protein concentration, adjustments in relative water content, modulation of photosynthetic pigment accumulation, modifications in antioxidant enzyme activity, and changes in proline and total soluble carbohydrate content [31]. In addition, the mRNA levels of some HSP70 gene family members significantly increased following exposure to salt stress in tomato [32]. Increased accumulation of Hsp70 proteins within the nucleus has been noted to enhance heat tolerance in transgenic *Arabidopsis* plants [33]. It seems that Hsp70s interact with and support specific transcription factors related to stress response, such as DREB2A, to boost rice’s resilience to abiotic factors [34]. Also, it was reported that abscisic acid, jasmonates, and ethylene predominantly exerted a negative influence on the expression of Hsp70 and the levels of the associated proteins. Conversely, cytokinin exhibited an up-regulatory effect on the majority of Hsp70s, positively influencing their abundance [25]. Hsp70 has the capacity to protect plant cells against programmed cell death triggered by elevated temperatures [35]. The quantity of members within distinct Hsp70 families varies, with counts ranging from 18 genes in *Arabidopsis thaliana* and 20 in *Solanum tuberosum*, to 30 in *Oryza sativa*, and up to 61 in *Nicotiana tabacum*, 61 genes in soybean, and 21 in pepper [36,37,38]. In the context of resistance to abiotic stresses, cytosolic/nuclear Hsp70s in *Arabidopsis thaliana* exhibited distinctive as well as overlapping functionalities [39]. The correlation between *HSP70* expression and thermotolerance in rice was robust, suggesting its potential as a prospective biomarker in forthcoming rice breeding initiatives [40]. It has been demonstrated that the heterologous expression of *PpHSP70* enhances rice tolerance to both heat and drought stress [41].

*Camelina sativa* L. Crantz (2*n* = 40, genome size approximately 782 Mb), a member of the Brassicaceae (Cruciferae) family, represents an economically significant crop originating from southeastern Europe and southwestern Asia [42]. *C. sativa*, characterized as a cultivation requiring minimal input and being environmentally sustainable, boasts a substantial seed oil content ranging from 36% to 47%. Consequently, it has attained global prominence as a widely cultivated oilseed crop [43,44]. Recently, computational techniques for comprehensive genomic analysis have been employed to identify novel proteins and genes. Despite the increasing popularity of omics technologies, there has been a notable absence of research focusing on the widespread identification and expression analysis of the HSP70 gene family in *Camelina sativa* under salinity stress. Therefore, this study undertook a comparative examination of Hsp70 proteins in *Camelina sativa* using broad-scale computational methodologies.

## 2. Results

### 2.1. Identification and Characterization of CsHSP70

In this comprehensive study, a total of 72 *CsHSP70* genes were identified and confirmed within *Camelina sativa*. These genes exhibited a remarkable diversity in their physicochemical properties, as detailed in Table S2. Notably, the protein length varied significantly, ranging from 151 amino acids to 2076 amino acids, while the exon number showed a wide range from 1 to 28. The molecular weight (MW) of these proteins also displayed considerable diversity. Furthermore, the isoelectric point (pI) for CsHSP70 proteins was found to fall within the range of 4.64 to 9.01, with a majority of proteins exhibiting a pI of less than 6.50. The grand average of hydropathy (GRAVY) value for CsHSP70 proteins was predicted to range between −0.939 and 0.126, with a notable observation that 96% of the proteins possessed a negative GRAVY index, indicating their hydrophilic nature. Subsequent subcellular localization predictions revealed that 31 CsHSP70 proteins were likely located in the cytoplasm, while 19 CsHSP70 proteins were predicted to be situated in the nucleus. Additionally, certain CsHSP70 proteins were anticipated to be active in the chloroplast, mitochondrion, extracellular space, and endomembrane system, further highlighting the diverse roles and functions of these proteins within *C. sativa*.

### 2.2. Phylogenetic Analysis

**Figure 1:**
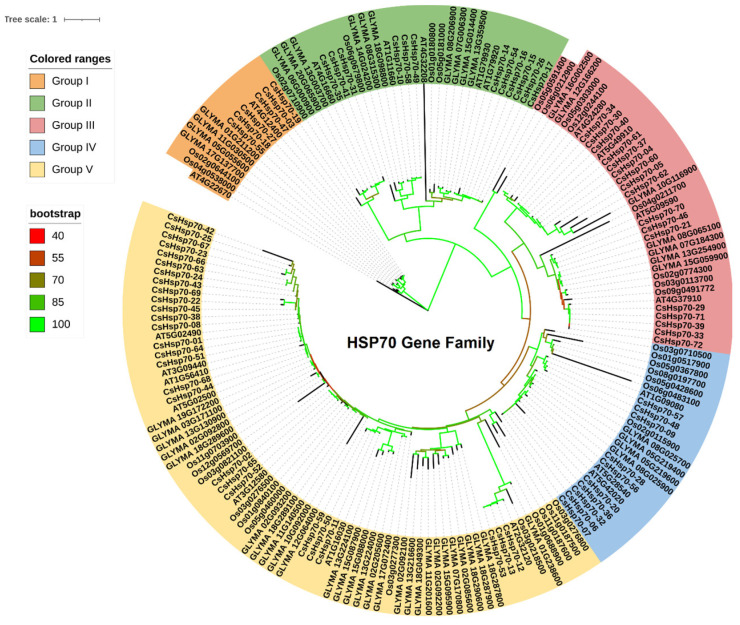
Evolution analysis of the HSP70 gene family based on the maximum likelihood method. Genes from *Camelina sativa* (starting with Cs), Arabidopsis thaliana (starting with At), *Glycine max* (starting with GLYMA), and *Oryza sativa* (starting with Os) were investigated.

The phylogenetic analysis conducted on the Hsp70 family members from *C. sativa* (CsHSP70), *O. sativa*, *A. thaliana*, and *G. max* resulted in the classification of these members into five main groups, as illustrated in Figure 1. Interestingly, the CsHSP70 family members exhibited the highest similarity with *Arabidopsis* orthologs, indicating a close evolutionary relationship between these species. The phylogenetic tree further highlighted the significant diversity present among CsHSP70 orthologs. Notably, group 5 contained the highest number of Hsp70 family members, while group 1 had the lowest. Group 1 comprised Hsp70s, which displayed a greater genetic distance from other orthologs, suggesting a distinct evolutionary path. The placement of rice Hsp70s, as a monocot model, indicated that the diversity within this family arose both before and after the divergence of monocots and dicots. Overall, this analysis provides valuable insights into the evolutionary history and relationships within the Hsp70 family, shedding light on the complex patterns of diversification and adaptation that have shaped these proteins over time.

### 2.3. Duplication Events

**Figure 2:**
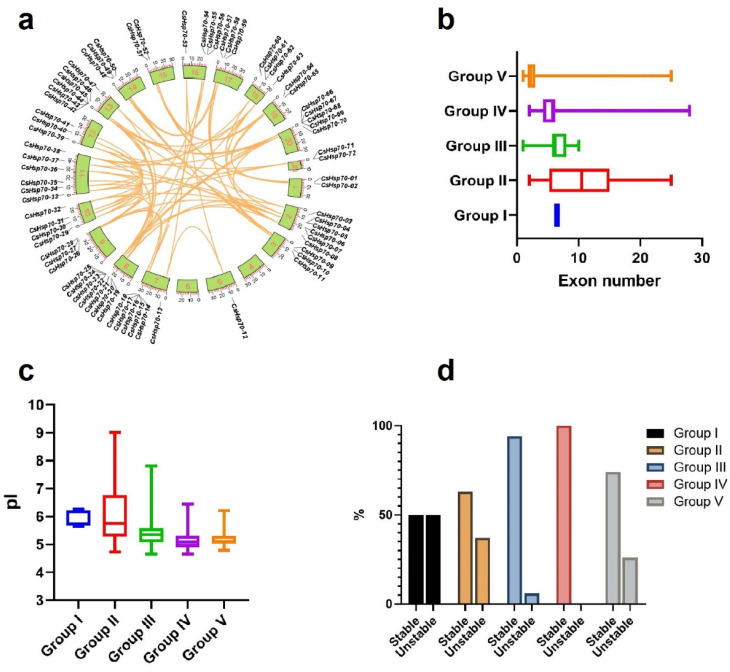
(**a**) Chromosomal distribution of *CsHsp70* genes in the camelina genome. Orange lines indicate duplicated genes. Comparison of CsHsp70 groups based on the number of exons (**b**), pI (**c**), and instability index (**d**).

The distribution of *CsHSP70* genes on the chromosomes reveals that, with the exception of chromosomes 4 and 6, all other camelina chromosomes contain at least one *CsHSP70* gene. Chromosome 8 stands out with seven genes, while chromosomes 2 and 13 each have six *CsHSP70* genes (Figure 2a). The presence of numerous segmental duplications between *CsHSP70* genes suggests that *CsHSP70* has been significantly impacted by evolutionary pressures. Phylogenetic analysis was conducted to compare different groups of *CsHSP70* genes based on exon number, isoelectric point (pI), and instability index. Group 1 exhibited less diversity in terms of exon number, whereas group 2 displayed greater diversity (Figure 2b). In terms of the isoelectric point, proteins in group 1 were mostly active under neutral conditions, while those in group 2 showed more variability (Figure 2c). Furthermore, based on the instability index, proteins in group 4 and the majority of those in group 3 were deemed stable, while an equal number of unstable proteins were predicted in group 1 compared to stable ones (Figure 2d).

### 2.4. Co-Expressed Transcription Factors (TFs) with CsHsp70 Genes

**Figure 3:**
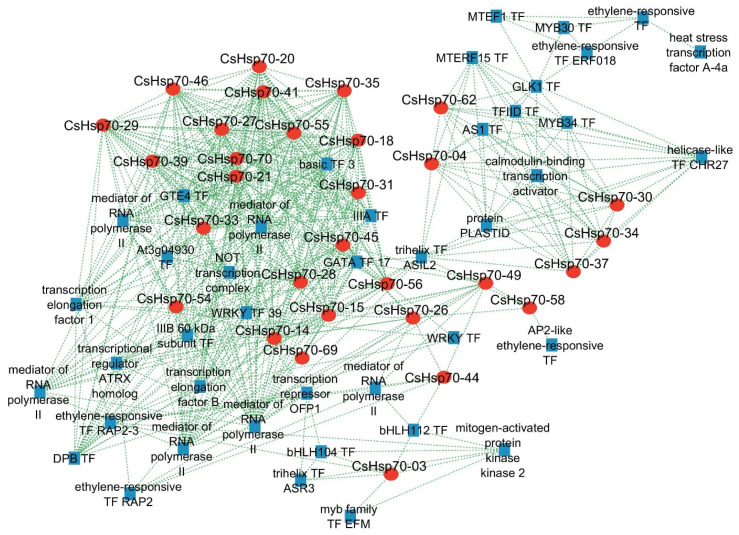
Co-expressed network between transcription factors (TFs) (blue dots) and *CsHsp70* genes (red dots).

**Figure 4:**
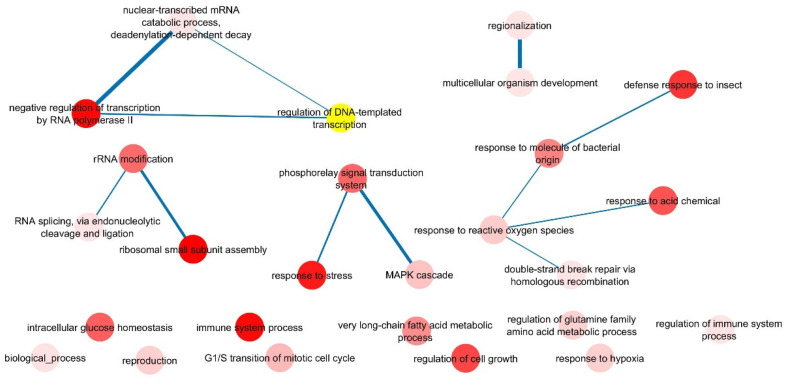
GO enrichment analysis of co-expressed network between transcription factors (TFs) and *CsHsp70* genes.

Considering the role of *Hsp70* in response to stress, an interaction network between transcription factors (TFs) and *CsHsp70* genes was drawn to better understand the regulatory pathways related to this gene family. The findings indicated that ethylene-responsive TFs, MYB TFs, and mediator of RNA polymerase II are highly co-expressed with *CsHsp70* genes (Figure 3). Additionally, MTERF, heat stress factor (HSF), helicase-like, trihelix, bHLH, ASA, GATA, WRKY TFs, and transcription elongation factor 1 were also found to be co-expressed with *CsHsp70* genes. Furthermore, a Gene Ontology (GO) enrichment analysis of the co-expression network elements revealed that key signaling terms such as phosphorelay signal transduction, response to stress (biotic and abiotic), response to reactive oxygen species (ROS), and MAPK cascade were significantly associated with CsHsp70 function (see Figure 4). The results also demonstrated that the expression patterns of *CsHsp70* genes are linked to TFs involved in cell growth, rRNA modification, and intracellular glucose homeostasis (see Figure 4). Overall, these findings shed light on the intricate relationship between *CsHsp70* genes and various TFs, providing valuable insights into the regulatory mechanisms underlying stress response in plants.

### 2.5. Expression Profile of CsHSP70 Family Members in Response to Abiotic Stresses

**Figure 5:**
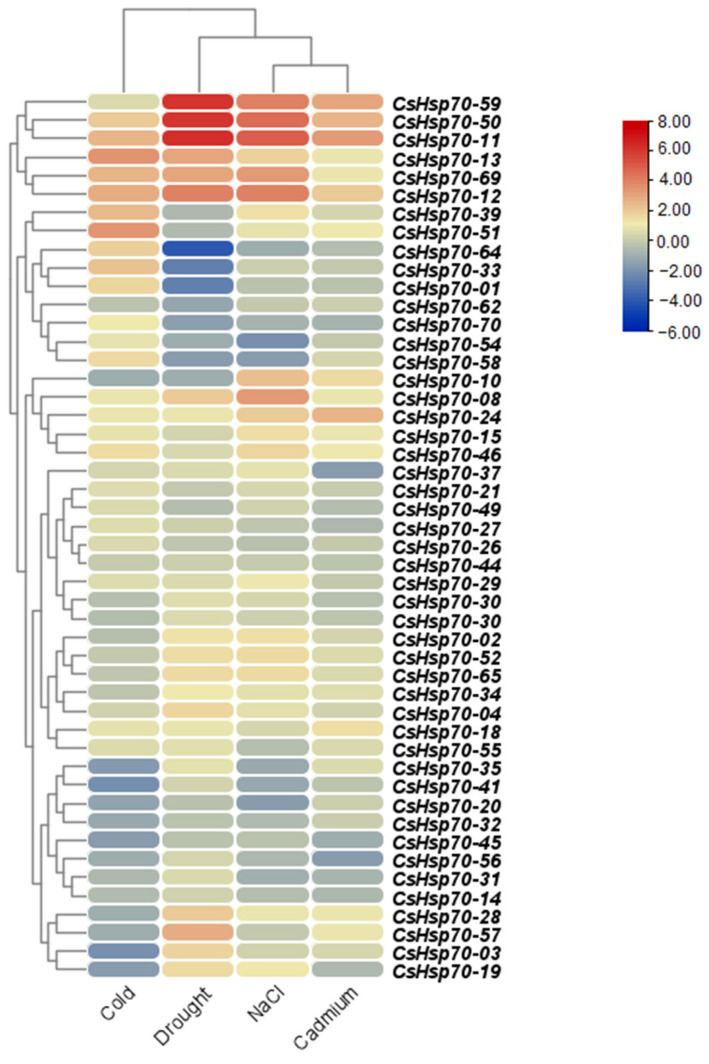
Expression profile of *CsHsp70* genes in response to abiotic stresses, including cold, drought, salinity and cadmium stress.

Transcriptome data analysis of camelina under abiotic stresses, including cold, drought, salinity, and cadmium stress, illustrated that *CsHsp70* genes have diverse expression patterns. Among these genes, *CsHsp70-13*, *CsHsp70-51*, and *CsHsp70-12* were more up-regulated in response to cold stress, while *CsHsp70-10*, *CsHsp70-35*, *CsHsp70-41*, *CsHsp70-03*, *CsHsp70-19*, *CsHsp70-45*, *CsHsp70-28*, *CsHsp70-57*, *CsHsp70-20*, and *CsHsp70-32* showed down-regulation (Figure 5). Four *CsHsp70* genes, *CsHsp70-59*, *CsHsp70-50*, *CsHsp70-11*, and *CsHsp70-12*, were up-regulated under drought, salt, and cadmium stresses. Furthermore, *CsHsp70-64*, *CsHsp70-33*, *CsHsp70-01*, and *CsHsp70-70* in response to drought stress, *CsHsp70-54*, *CsHsp70-58*, and *CsHsp70-20* in response to salinity stress, and *CsHsp70-37*, *CsHsp70-56*, and *CsHsp70-70* in response to cadmium stress were more down-regulated.

### 2.6. Expression Profile of CsHSP70 Family Members in Organs and Developmental Stages

**Figure 6:**
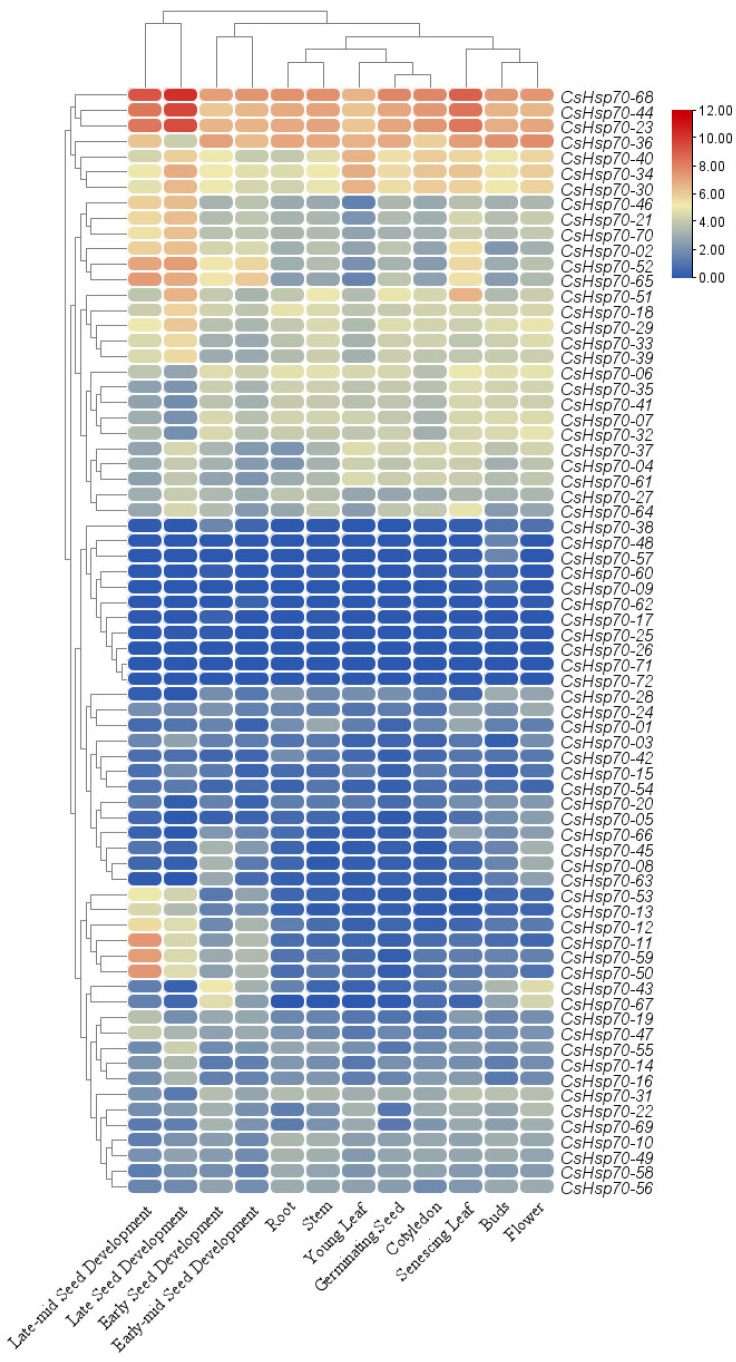
Expression profile of *CsHsp70* genes in different tissues and developmental stages of camelina.

The expression patterns of *CsHSP70* genes in various organs and developmental stages reveal that *CsHSP70* genes are active in all organs and play a crucial role in growth and development (see Figure 6). Among the *CsHSP70* genes studied, *CsHSP70-68*, *CsHSP70-44*, *CsHSP70-36*, and *CsHSP70-23* exhibited high expression levels across all tissues and developmental stages (see Figure 6). However, certain *CsHSP70* genes displayed tissue-specific expression patterns. For example, *CsHSP70-11* was found to be more highly expressed in senescing leaves and during late seed development. Furthermore, *CsHSP70-11*, *CsHSP70-59*, and *CsHSP70-50* were observed to have increased expression levels during the late-mid seed development stages of camelina. Interestingly, the expression patterns of *CsHSP70* genes in bud and flower tissues were similar, as were the patterns observed between stem and root tissues.

### 2.7. Relative Expression of CsHSP70 in Response to Salinity

**Figure 7:**
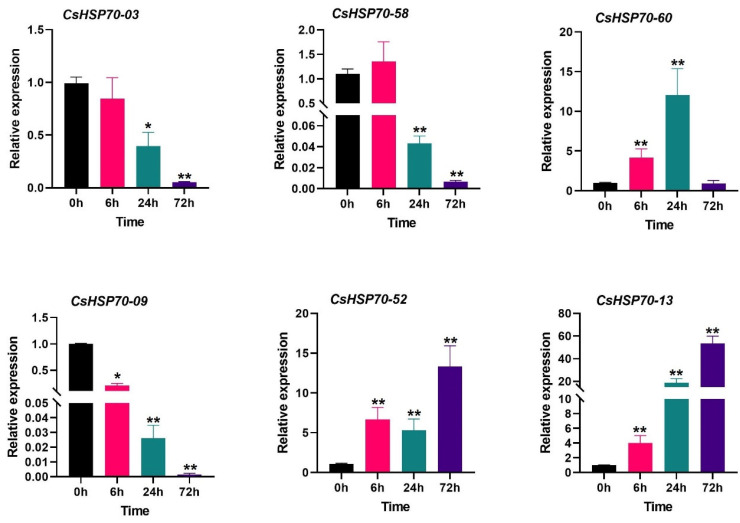
Expression profile of *CsHSP70* genes in response to salinity stress at different time points. * and ** indicate significant differences between treatments and the control sample (0 h) at the *p*-value < 0.05 and *p*-value < 0.01 level, respectively, based on Student’s *t*-test.

The expression levels of *CsHSP70* genes were analyzed using quantitative PCR (qPCR) in response to salt stress, as shown in Figure 7. *CsHSP70-03* and *CsHSP70-58* exhibited a similar expression pattern, both being down-regulated after 24 h of exposure to salt stress. Furthermore, their expression levels were significantly decreased after 72 h of salinity stress. On the other hand, *CsHSP70-09* was rapidly induced by salt stress, but its expression showed a substantial down-regulation after 72 h. In contrast, three *CsHSP70* genes, namely, *CsHSP70-60*, *CsHSP70-52*, and *CsHSP70-13*, were up-regulated in response to salinity in the short term. *CsHSP70-60* displayed a high expression level after 24 h, while *CsHSP70-52* and *CsHSP70-13* were more induced after 72 h of salt stress. These findings suggest that *CsHSP70* genes play diverse roles in response to salinity stress.

### 2.8. Docking Analysis Between CsHsp70 Proteins and Hormones

**Figure 8:**
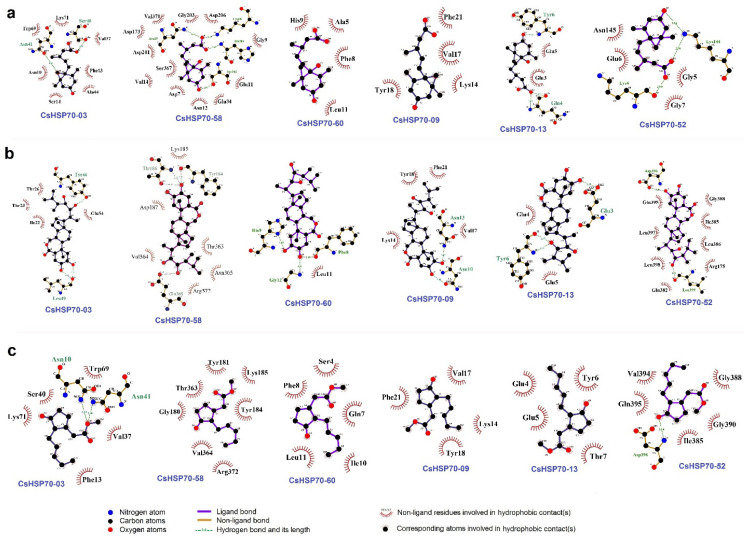
Docking analysis between CsHsp70 proteins and ABA (**a**), BR (**b**), and MeJA (**c**). Candidate proteins were selected based on phylogenetic analysis.

Docking analysis was conducted to investigate the potential interactions between candidate CsHsp70 proteins and abscisic acid (ABA), brassinosteroid (BR), and MeJA. The results, illustrated in Figure 8, revealed both ligand and non-ligand interactions between the hormones and CsHsp70 proteins, showcasing the diverse nature of these interactions. Specifically, CsHsp70 proteins were found to predominantly interact with ABA through amino acids ASN, Lys, Glu, and Val (Figure 8a). Additionally, amino acids Leu, Glu, Tyr, and Thr were identified as key players in the binding of CsHsp70 proteins to BR (Figure 8b). Furthermore, amino acids Val, Lys, Phe, Gly, Glu, and Thr were highlighted as crucial in the interaction between CsHsp70 proteins and MeJA (Figure 8c). Interestingly, it was observed that CsHsp70-09 and CsHsp70-13 shared the same binding regions for ABA, MeJA, and BR molecules, indicating potential similarities in their interactions with these hormones.

### 2.9. Upstream Analysis of CsHsp70 Genes

**Figure 9:**
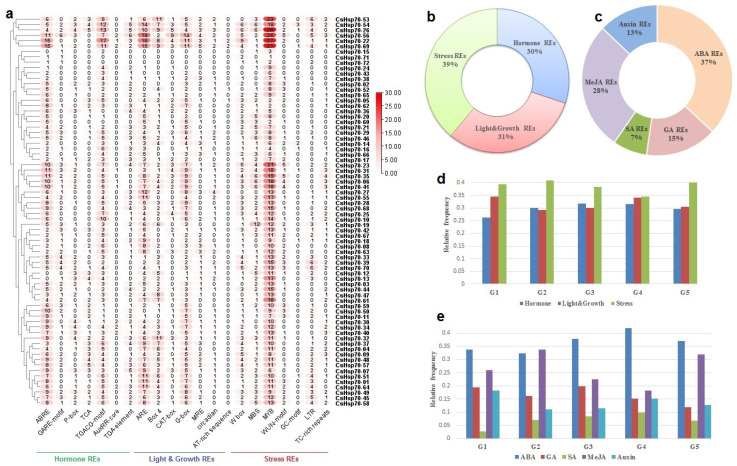
Predicted cis-regulatory elements in the promoter site of CsHsp70 gene family members. (**a**) Statistics of the number of each *cis*-regulatory elements in the promoter region of the *CsHsp70* gene. (**b**) Frequency percentage of regulatory elements (REs) according to their function in three groups, namely, hormone REs, growth and light REs, and stress REs. (**c**) Frequency percentage of regulatory elements (REs) related to hormone responsiveness. (**d**) Comparison between *CsHsp70* groups according to the frequency of identified cis-regulatory elements. (**e**) Comparison between *CsHsp70* groups according to the frequency of identified *cis*-regulatory elements related to hormone responsiveness.

To gain insight into the regulatory mechanisms influencing gene function, we conducted an investigation into the upstream region of *CsHsp70* genes. Our findings revealed variations in the frequency of *cis*-regulatory elements among *CsHsp70* genes, potentially impacting their functionality (Figure 9a; Table S3). Specifically, *cis*-regulatory elements associated with abscisic acid response (ABREs), MeJA-responsiveness (TGACG-motif), and stress response, such as MYB transcription factors, were prominently present in the promoter region of *CsHsp70* genes (Figure 9a). These identified cis elements were categorized based on their functions, with a majority falling under the stress response category (Figure 9b). Furthermore, when examining hormone response elements, *cis*-regulatory elements linked to abscisic acid (ABA, 37%) and methyl jasmonate (MeJA, 28%) were found to be recurrent in the promoter region of *CsHsp70* genes (Figure 9c). By comparing the phylogeny groups of *CsHsp70* based on the function of *cis*-elements, we observed a prevalence of stress response elements across all groups (Figure 9d). Additionally, a comparison of phylogeny groups based on hormone response *cis*-elements revealed a higher frequency of elements associated with ABA response in all groups (except group 2), while members of group 2 exhibited a higher frequency of elements related to MeJA response (Figure 9e). Overall, our study sheds light on the diverse regulatory mechanisms influencing *CsHsp70* genes, with a particular emphasis on stress response elements and hormone-related *cis*-regulatory elements.

## 3. Discussion

The gene encoding HSP70, a crucial cellular chaperone, exhibits increased expression at both the mRNA and protein levels in plant cells exposed to various stresses. Evidence suggests that the retention of memory from previous stress exposure may be linked to the activation of signaling pathways associated with HSP70. Hsp70 proteins play vital roles in responding to abiotic stress by aiding in numerous protein-folding processes. However, the HSP70 gene family of *C. sativa* (CsHsp70) has not been previously identified or characterized. In our current study, we identified 72 CsHSP70 family members within the genome of *C. sativa*. It is worth noting that the number of genes within the Hsp70 family varies among plant species. For instance, *Arabidopsis* has 18 members [45], while soybean and *Chrysanthemum lavandulifolium* have 61 and 83 genes, respectively [46]. This discrepancy in gene numbers suggests that factors such as ploidy level and genome size significantly influence the size of a gene family [47]. The total of CsHSP70 family members may be linked to the camelina adaptability and ecological environments of varied plant species, allowing for insightful observations into their features.

Evolutionary pressures, such as duplication events, have played a significant role in the expansion of gene family members in plants. In the current study, a high number of segmental-duplication events were identified in *CsHSP70* genes, indicating a substantial expansion of *CsHSP70* under these evolutionary pressures. Additionally, variations in physicochemical characteristics were observed among CsHSP70 family members, suggesting that these genes have undergone changes throughout evolution, leading to functional diversity [47,48]. Furthermore, CsHSP70 family members and their orthologs were categorized into five distinct groups, revealing significant variation both within and between species. Notably, members of group 2 in the phylogenetic tree exhibited wide variations in traits such as isoelectric point (pI) and exon number. The analysis of gene structure and phylogenetic trees has proven to be invaluable in understanding the evolutionary relationships among genes [49]. Genes that are closely related in terms of phylogeny often share similar properties or functions, and are typically consistent in their subcellular locations [50,51]. The number of exons in a gene can impact gene expression speed and isoform diversity, highlighting the importance of this trait in understanding the functional diversity of CsHSP70 family members [52]. Furthermore, the number of introns present in a gene is often influenced by transcriptional regulation [47,53]. Each of these genes contains one intron and two exons, with the proteins they encode being localized in the cytoplasm, suggesting a potential similarity in function. It has been noted that genes with a higher number of introns tend to respond more slowly to stresses, whereas genes with fewer introns are more sensitive to stressors [54]. This information highlights the importance of gene structure and phylogenetic analysis in understanding gene evolution and function.

The subcellular localization prediction indicates that the majority of CsHsp70 proteins are active in both the cytoplasm and nucleus. Additionally, these proteins function within organelles such as chloroplasts and mitochondria. This extensive distribution of CsHsp70s across various cellular components enhances their efficacy, ultimately mitigating stress-induced damage. Furthermore, results indicate that *CsHsp70* genes may have a stronger interaction with MYB transcription factors, the mediator of RNA polymerase II, MTERF transcription factors, and heat stress factors (HSFs) in camelina. MYBs are known to be highly active transcription factors in response to unfavorable environmental conditions. Recent research in rice has shown that the activation of Hsp70B is mediated by MYB21 [55]. When rice plants experience heat stress, the temperature shock transcription factor becomes activated. This factor, along with RNA polymerase II, plays a crucial role in inducing *HSP* genes [56]. Furthermore, a GO enrichment analysis of co-expression networks has revealed that *CsHsp70* genes and their co-expressed transcription factors work together in important signaling pathways. These pathways include responses to reactive oxygen species (ROS), various types of stress (both biotic and abiotic), and the MAPK cascade associated with environmental stresses. Additionally, the promoter regions of *CsHsp70* genes contain binding sites for transcription factors and response elements related to ABA and MeJA. This suggests that these genes are involved in signaling pathways that are dependent on hormones and stress. Differences were observed among the phylogenetic groups of CsHsp70 based on the frequency of cis elements. Specifically, MeJA-responsive elements were more prevalent in members of group 2. Previous research by Duan et al. (2011) [57] indicated that HSP70 is rapidly induced after MeJA application in wheat. The present study utilized docking analysis to uncover non-ligand interactions between CsHsp70 proteins and ABA, BR, and MeJA. These results indicate a potential connection between CsHsp70s and signaling pathways related to ABA and MeJA responses.

The expression levels of *CsHsp70* genes varied across various organs and developmental stages of camelina, highlighting the diverse roles of these genes in the plant’s growth and development. The *CsHsp70* genes are expressed in various tissues, with some showing tissue-specific expression patterns. Specifically, *CsHsp70-11*, *CsHsp70-59*, and *CsHsp70-50* exhibit specific expression during the late-mid stages of seed development in camelina. Previous studies have also documented the tissue-specific expression of *HSP70* genes in plants, such as the tea *CsHSP70s* [58,59]. Moreover, the expression levels of *HSP70-11/-16* homologous genes in pepper were sharply different in tissues [19]. Furthermore, the transcriptome profile of camelina showed that *CsHsp70* genes are involved in response to adverse conditions such as cold, drought, salinity, and cadmium stress.

Research has shown that the overexpression of *HSP70* genes can enhance tolerance to high temperatures, water scarcity, and salinity stress in Nicotiana and *Arabidopsis* plants [60,61,62,63]. Furthermore, studies have revealed that the *PtHsp70* genes in Populus exhibit varying levels of expression in drought-tolerant and -sensitive species [64], while barley *Hsp70* genes are more strongly induced in response to drought stress [65]. Additionally, research has shown that many *HSP70* genes in grapes are induced by low temperatures [66]. The current study focused on investigating four *CsHsp70* genes, specifically, *CsHsp70-59*, *CsHsp70-50*, *CsHsp70-11*, and *CsHsp70-12*, and their involvement in responding to abiotic stresses. These genes are potential candidates for further molecular functional research to elucidate their role in the common signaling pathway associated with abiotic stress responses. Through qRT-PCR analysis, it was revealed that *CsHsp70-60*, *CsHsp70-52*, and *CsHsp70-13* were up-regulated, while *CsHsp70-03*, *CsHsp70-58*, and *CsHsp70-09* showed down-regulation in response to salinity stress. Furthermore, the *CsHsp70* genes can be categorized as late-response elements to salinity stress. The presence of NaCl stress not only includes the challenge of salinity stress but also the risk of alkaline stress. Crop production suffers from the harmful impact of both high salinity and elevated pH levels caused by saline-alkaline stress [67,68]. It appears that saline-alkaline stress triggers a shock and activates downstream cellular signaling pathways that influence the expression levels of *CsHsp70* genes. The expression pattern of the *CsHsp70* genes in response to salinity stress in qPCR analysis was not consistent with the data obtained from RNA-seq datasets. Environmental conditions (seedling age, temperature conditions, light intensity, etc.) and plant genotype can affect the discrepancy between these data, although the accuracy of qPCR data is higher than that of RNA-seq data. Overall, this study sheds light on the potential roles of specific *CsHsp70* genes in responding to abiotic stresses, particularly salinity stress, and highlights the importance of further research to fully understand their functions in stress response pathways.

## 4. Materials and Methods

### 4.1. Identification and Characterization

To identify the Hsp70 gene family member in *Camelina sativa* (CsHsp70), the Hsp70 proteins from the dicot model, *Arabidopsis thaliana*, were used as queries against camelina genome using the Blastp tool of the Ensembl Plants database [69]. All recognized proteins were checked by the Pfam for having a conserved domain. The coding sequences and cDNA sequences of confirmed proteins were download from the Ensembl Plants database. Additionally, the ProtParam tool [70] was applied to predict the physiochemical properties such as molecular weight (MW), isoelectric point (pI), instability index, and grand average of hydropathicity (GRAVY).

### 4.2. Phylogenetic Analysis and Prediction Duplication Events

The amino acid sequences of the Hsp70 family from *Arabidopsis thaliana, Glycine max*, and *Oryza sativa,* along with CsHSP70 proteins, were used to construct a phylogeny tree. In the first step, multiple alignment analysis was applied using Clustal Omega [71] and then the aligned sequences were imported to the IQ-TREE web server [72]. Finally, the phylogeny tree was constructed using the maximum likelihood (ML) method. The iTOL (Interactive Tree Of Life) [73] was applied to visualize the phylogeny tree of the Hsp70 gene family. Additionally, the duplication events of *CsHsp70* genes were predicted based on similarity. In the first step, the coding sequences of *CsHsp70* genes were analyzed using the multiple sequence alignment tool of Mega X [74] and pairs of *CsHsp70* genes with an identity more than 0.85 were recognized as duplicated genes. In addition, the tandem and segmental duplication were identified based on the location of duplicated genes.

### 4.3. Promoter Analysis

The upstream sequences (1500 bp), as the promoter region, of *CsHsp70* genes were downloaded from the Ensembl Plants database and then these regions were analyzed by the PlantCARE database [75] to screen the cis-regulatory elements related to hormones, light and growth, and stress responses.

### 4.4. Expression Profile of CsHsp70 Genes

RNA-seq expression data of *Camelina sativa* genes were collected and analyzed based on the method described by Pertea et al. [76]. Briefly, fastq files were assessed by the FastQC software v.0.12.1 (https://www.bioinformatics.babraham.ac.uk/projects/fastqc/, accessed on 25 February 2024). The adapter and low-quality regions were removed by Trimmomatic. Alignment of RNA-seq data and reference genome was conducted by HISAT, and HTSeq-count [77] calculated the read count for genes. Differentially expressed gene analysis was performed by the R package NOISeq [78], accessed on 28 February 2024. Gene expression data were visualized by the pheatmap R package, accessed on 28 February 2024.

### 4.5. Bi-Clustering Analysis

Co-expressed transcription factors (TFs) with CsHsp70 genes were identified by the R package QUBIC [79]. A seed containing *CsHsp70* genes was extracted from expression data in different conditions and tissues. We ran QUBIC on the seed to build an initial bicluster, and this was then run on whole genes to add co-expressed genes to the bicluster. TF IDs of *Camelina sativa* were obtained from NCBI and were searched in co-expressed gene IDs with *CsHsp70* genes.

### 4.6. GO Enrichment Analysis of Co-Expressed TFs

Gene IDs of co-expressed TFs with *CsHsp70*s were converted to GeneBank Protein Accession by bioDBnet (https://biodbnet-abcc.ncifcrf.gov/, accessed on 28 February 2024), and protein sequences of co-expressed TFs were downloaded from NCBI in fasta format. To functional annotation of co-expressed TFs, protein sequences were uploaded to Eggnog-mapper [80], accessed on 10 March 2024. GO terms were mapped and visualized by the REVIGO tool [81], accessed on 11 March 2024. Finally, a co-expression network was drawn by Cytoscape v. 3.9.0 [82].

### 4.7. Molecular Docking Analysis

The interaction of phytohormones (brassinosteroid, abscisic acid, and methyl jasmonate) and CsHsp70 was investigated by docking analysis. We selected five of the CsHsp70s (one member of each phylogenetic group). The 3D structure of CsHsp70 proteins as receptors was predicted by trRosetta server [83], and the 3D structure of phytohormones as ligands was downloaded from PubChem (https://pubchem.ncbi.nlm.nih.gov/, accessed on 15 March 2024). Receptors and ligands were converted to pdbqt format by AutoDockTool-1.5.7 and molecular docking was conducted using AutoDock Vina [84]. Docking results were validated by the PLIP web server [85] and visualized by LigPlot [86], accessed on 15 March 2024.

### 4.8. Plant Materials and Treatments

In the present study, expression levels of six candidate *CsHsp70* genes were investigated in response to salinity stress. The seeds were sterilized by a 3% sodium hypochlorite solution for 2 min, then rinsed twice with sterile water. Seeds were grown in pots containing perlite and peat moss (1:2), under a 16 h light period and normal temperature (24 ± 2 °C), and with irrigation once every three days. When the seedlings were five weeks old, salinity stress was applied by irrigation with salt solution (200 mM NaCl). After applying the salinity stress, the leaves from different seedlings were harvested at different times (6, 24, and 72 h) and placed in liquid nitrogen. In this experiment, three independent biological replicates were used.

### 4.9. qPCR Analysis

An RNX kit (Sinaclon, Tehran, Iran) was used to extract RNA based on the manufacturer’s protocols. Then, complementary DNA (cDNA) was made using a reverse transcriptase kit (Roche, Mannheim, Germany) according to the manufacturer’s protocols. The expression patterns of candidate *CsHsp70* genes were investigated using a Maxima SYBR Green/ROX qPCR Master Mix kit (Thermo Fisher, Illkirch-Graffenstaden, France) by ABI Step One, based on the manufacturer’s protocols. Six *CsHsp70* genes were selected based on evolutionary analysis, *CsHsp70-03* from group 1, *CsHsp70-58* from group 2, *CsHsp70-60* from group 3, *CsHsp70-09* from group 4, and *CsHsp70-52* and *CsHsp70-13* from group 5. Additionally, the *actin-2* gene (Csa15g026420) was used as a reference gene. Specific primers were designed using the online tool Primer Blast (Table S1). The relative expression levels of candidate *CsHsp70* genes were calculated using the delta–delta Ct method based on raw data [87].

## 5. Conclusions

In this analysis, we addressed the CsHsp70 gene family and examined aspects such as gene structure, protein properties, evolutionary aspects, functional sites, upstream control system, interaction with transcription factors, and hormone interaction regions. Also, the function of these genes in response to salt stress was investigated. The results showed that CsHsp70 cooperates with the hormones of abscisic acid and methyl jasmonate in response to stresses, and participates in the regulation of downstream signaling pathways related to environmental stresses by interacting with transcription factors. The expression profile of *CsHsp70* genes indicated that these genes are involved in the response to salt stress. The study of CsHsp70 genes in different plant species, including camelina, is very important for understanding the molecular mechanisms underlying plant growth and adaptation to environmental challenges. This knowledge can be used to improve plant breeding programs, increase plant resistance, and develop strategies for oilseed production under harsh environmental conditions.

## Data Availability

The original contributions presented in the study are included in the article/Supplementary Material, further inquiries can be directed to the corresponding author.

## Supplementary Materials

The following supporting information can be downloaded at: https://www.mdpi.com/article/10.3390/plants13233410/s1, Table S1: List of primers used in qPCR analysis; Table S2: List of CsHsp70 family members and their physicochemical properties; Table S3: Distribution of cis-regulatory elements in promoter region of CsHsp70 genes.

## References

[1] Timperio A.M., Egidi M.G., Zolla L. (2008). Proteomics Applied on Plant Abiotic Stresses: Role of Heat Shock Proteins (HSP). J. Proteom..

[2] Wang W., Vinocur B., Shoseyov O., Altman A. (2004). Role of Plant Heat-Shock Proteins and Molecular Chaperones in the Abiotic Stress Response. Trends Plant Sci..

[3] Pan X., Zheng Y., Lei K., Tao W., Zhou N. (2024). Systematic Analysis of Heat Shock Protein 70 (HSP70) Gene Family in Radish and Potential Roles in Stress Tolerance. BMC Plant Biol..

[4] Abasi F., Raja N.I., Mashwani Z.-R., Ehsan M., Ali H., Shahbaz M. (2024). Heat and Wheat: Adaptation Strategies with Respect to Heat Shock Proteins and Antioxidant Potential; an Era of Climate Change. Int. J. Biol. Macromol..

[5] Abou-Deif M.H., Rashed M.A.-S., Khalil K.M., Mahmoud F.E.-S. (2019). Proteomic Analysis of Heat Shock Proteins in Maize (*Zea mays* L.). Bull. Natl. Res. Cent..

[6] Sung D., Kaplan F., Guy C.L. (2001). Plant Hsp70 Molecular Chaperones: Protein Structure, Gene Family, Expression and Function. Physiol. Plant..

[7] Al-Whaibi M.H. (2011). Plant Heat-Shock Proteins: A Mini Review. J. King Saud Univ.-Sci..

[8] Izumi M. (2019). Heat Shock Proteins Support Refolding and Shredding of Misfolded Proteins. Plant Physiol..

[9] Divya K., Bhatnagar-Mathur P., Sharma K.K., Reddy P.S. (2019). Heat Shock Proteins (Hsps) Mediated Signalling Pathways during Abiotic Stress Conditions. Plant Signaling Molecules.

[10] McLoughlin F., Basha E., Fowler M.E., Kim M., Bordowitz J., Katiyar-Agarwal S., Vierling E. (2016). Class I and II Small Heat Shock Proteins Together with HSP101 Protect Protein Translation Factors during Heat Stress. Plant Physiol..

[11] Nitnavare R.B., Yeshvekar R.K., Sharma K.K., Vadez V., Reddy M.K., Reddy P.S. (2016). Molecular Cloning, Characterization and Expression Analysis of a Heat Shock Protein 10 (Hsp10) from *Pennisetum glaucum* (L.), a C4 Cereal Plant from the Semi-Arid Tropics. Mol. Biol. Rep..

[12] Zhao P., Wang D., Wang R., Kong N., Zhang C., Yang C., Wu W., Ma H., Chen Q. (2018). Genome-Wide Analysis of the Potato Hsp20 Gene Family: Identification, Genomic Organization and Expression Profiles in Response to Heat Stress. BMC Genom..

[13] Song K., Yim W.C., Lee B.-M. (2017). Expression of Heat Shock Proteins by Heat Stress in Soybean. Plant Breed. Biotechnol..

[14] Gao P., Lu M.X., Pan D.D., Du Y.Z. (2020). Characterization of an Inducible HSP70 Gene in Chilo Suppressalis and Expression in Response to Environmental and Biological Stress. Cell Stress Chaperones.

[15] Wang T.Y., Wu J.R., Duong N.K.T., Lu C.A., Yeh C.H., Wu S.J. (2021). HSP70-4 and Farnesylated AtJ3 Constitute a Specific HSP70/HSP40-Based Chaperone Machinery Essential for Prolonged Heat Stress Tolerance in Arabidopsis. J. Plant Physiol..

[16] Alderson T.R.R., Kim J.H.H., Markley J.L.L. (2016). Dynamical Structures of Hsp70 and Hsp70-Hsp40 Complexes. Structure.

[17] Montero-Barrientos M., Hermosa R., Cardoza R.E., Gutiérrez S., Nicolás C., Monte E. (2010). Transgenic Expression of the Trichoderma Harzianum Hsp70 Gene Increases Arabidopsis Resistance to Heat and Other Abiotic Stresses. J. Plant Physiol..

[18] Clerico E.M., Meng W., Pozhidaeva A., Bhasne K., Gierasch L.M. (2019). Hsp70 Molecular Chaperones: Multifunctional Allosteric Holding and Unfolding Machines. Biochem. J..

[19] Guo M., Liu J.H., Ma X., Zhai Y.F., Gong Z.H., Lu M.H. (2016). Genome-Wide Analysis of the Hsp70 Family Genes in Pepper (*Capsicum annuum* L.) and Functional Identification of CaHsp70-2 Involvement in Heat Stress. Plant Sci..

[20] Wen F., Wu X., Li T., Jia M., Liu X., Li P., Zhou X., Ji X., Yue X. (2017). Genome-Wide Survey of Heat Shock Factors and Heat Shock Protein 70s and Their Regulatory Network under Abiotic Stresses in Brachypodium Distachyon. PLoS ONE.

[21] Khan M., Jannat A., Munir F., Fatima N., Rabia A. (2020). Biochemical and Molecular Mechanisms of Abiotic Stress Tolerance. Plant Ecophysiology and Adaptation under Climate Change: Mechanisms and Perspectives II: Mechanisms of Adaptation and Stress Amelioration.

[22] Yer E.N., Baloglu M.C., Ziplar U.T., Ayan S., Unver T. (2016). Drought-Responsive Hsp70 Gene Analysis in Populus at Genome-Wide Level. Plant Mol. Biol. Rep..

[23] Sable A., Agarwal S.K. (2018). Plant Heat Shock Protein Families: Essential Machinery for Development and Defense. J. Biol. Sci. Med..

[24] Lin B.L., Wang J.S., Liu H.C., Chen R.W., Meyer Y., Barakat A., Delseny M. (2001). Genomic Analysis of the Hsp70 Superfamily in Arabidopsis Thaliana. Cell Stress Chaperones.

[25] Berka M., Kopecká R., Berková V., Brzobohatý B., Černý M. (2022). Regulation of Heat Shock Proteins 70 and Their Role in Plant Immunity. J. Exp. Bot..

[26] Willems P., Horne A., Van Parys T., Goormachtig S., De Smet I., Botzki A., Van Breusegem F., Gevaert K. (2019). The Plant PTM Viewer, a Central Resource for Exploring Plant Protein Modifications. Plant J..

[27] Nitika, Porter C.M., Truman A.W., Truttmann M.C. (2020). Post-Translational Modifications of Hsp70 Family Proteins: Expanding the Chaperone Code. J. Biol. Chem..

[28] Ul Haq S., Khan A., Ali M., Khattak A.M., Gai W.X., Zhang H.X., Wei A.M., Gong Z.H. (2019). Heat Shock Proteins: Dynamic Biomolecules to Counter Plant Biotic and Abiotic Stresses. Int. J. Mol. Sci..

[29] Sarkar N.K., Kundnani P., Grover A. (2013). Functional Analysis of Hsp70 Superfamily Proteins of Rice (*Oryza sativa*). Cell Stress Chaperones.

[30] Fragkostefanakis S., Röth S., Schleiff E., Scharf K. (2015). Prospects of Engineering Thermotolerance in Crops through Modulation of Heat Stress Transcription Factor and Heat Shock Protein Networks. Plant J..

[31] Anaraki Z.E., Tafreshi S.A.H., Shariati M. (2018). Transient Silencing of Heat Shock Proteins Showed Remarkable Roles for HSP70 during Adaptation to Stress in Plants. Environ. Exp. Bot..

[32] Vu N.T., Nguyen N.B.T., Ha H.H., Nguyen L.N., Luu L.H., Dao H.Q., Vu T.T., Huynh H.T.T., Le H.T.T. (2023). Evolutionary Analysis and Expression Profiling of the HSP70 Gene Family in Response to Abiotic Stresses in Tomato (*Solanum lycopersicum*). Sci. Prog..

[33] Koizumi S., Ohama N., Mizoi J., Shinozaki K., Yamaguchi-Shinozaki K. (2014). Functional Analysis of the Hikeshi-like Protein and Its Interaction with HSP70 in Arabidopsis. Biochem. Biophys. Res. Commun..

[34] Wang Q., Guan Æ.Y., Wu Æ.Y., Chen H., Chen Æ.F., Chu Æ.C. (2008). Overexpression of a Rice OsDREB1F Gene Increases Salt, Drought, and Low Temperature Tolerance in Both Arabidopsis and Rice. Plant Mol. Biol..

[35] Lin J.S., Kuo C.C., Yang I.C., Tsai W.A., Shen Y.H., Lin C.C., Liang Y.C., Li Y.C., Kuo Y.W., King Y.C. (2018). MicroRNA160 Modulates Plant Development and Heat Shock Protein Gene Expression to Mediate Heat Tolerance in Arabidopsis. Front. Plant Sci..

[36] Liu J., Pang X., Cheng Y., Yin Y., Zhang Q., Su W., Hu B., Guo Q., Ha S., Zhang J. (2018). The Hsp70 Gene Family in Solanum Tuberosum: Genome-Wide Identification, Phylogeny, and Expression Patterns. Sci. Rep..

[37] Song Z., Pan F., Lou X., Wang D., Yang C., Zhang B., Zhang H. (2019). Genome-Wide Identification and Characterization of Hsp70 Gene Family in Nicotiana Tabacum. Mol. Biol. Rep..

[38] Liang Z., Li M., Liu Z., Wang J. (2019). Genome-Wide Identification and Characterization of the Hsp70 Gene Family in Allopolyploid Rapeseed (*Brassica napus* L.) Compared with Its Diploid Progenitors. PeerJ.

[39] Leng L., Liang Q., Jiang J., Zhang C., Hao Y., Wang X., Su W. (2017). A Subclass of HSP70s Regulate Development and Abiotic Stress Responses in Arabidopsis Thaliana. J. Plant Res..

[40] Ali M.K., Azhar A., us Salam E., Galani S. (2017). Differential Expression of Molecular Chaperon (HSP70) and Antioxidant Enzymes: Inducing Thermotolerance in Rice (*Oryza sativa* L.). Pak. J. Bot..

[41] Kou S.Y., Wu Z.G., Li H.Y., Chen X., Liu W.H., Yuan P.R., Zhu Z.H., Yang X., Li H.H., Huang P. (2023). Heterologous Expression of Heat-Shock Protein PpHSP70 Improves High Temperature and Drought Tolerance in Rice. Plant Stress.

[42] Luo Z., Tomasi P., Fahlgren N., Abdel-Haleem H. (2019). Genome-Wide Association Study (GWAS) of Leaf Cuticular Wax Components in Camelina Sativa Identifies Genetic Loci Related to Intracellular Wax Transport. BMC Plant Biol..

[43] Luo T., Song Y., Gao H., Wang M., Cui H., Ji C., Wang J., Yuan L., Li R. (2022). Genome-Wide Identification and Functional Analysis of Dof Transcription Factor Family in Camelina Sativa. BMC Genom..

[44] Moser B.R. (2012). Biodiesel from Alternative Oilseed Feedstocks: Camelina and Field Pennycress. Biofuels.

[45] Sarwat M., Tuteja N. (2017). Hormonal Signaling to Control Stomatal Movement during Drought Stress. Plant Physiol..

[46] Yin M., Hu R., Song A., Guan Z., Chen F., Jiang J. (2023). Genome-Wide Identification and Expression Analysis of HSP70 Gene Family in Chrysanthemum Lavandulifolium under Heat Stress. Horticulturae.

[47] Yaghobi M., Heidari P. (2023). Genome-Wide Analysis of Aquaporin Gene Family in Triticum Turgidum and Its Expression Profile in Response to Salt Stress. Genes.

[48] Hashemipetroudi S.H., Arab M., Heidari P., Kuhlmann M. (2023). Genome-Wide Analysis of the Laccase (LAC) Gene Family in Aeluropus Littoralis: A Focus on Identification, Evolution and Expression Patterns in Response to Abiotic Stresses and ABA Treatment. Front. Plant Sci..

[49] Wang Y., Hua X., Xu J., Chen Z., Fan T., Zeng Z., Wang H., Hour A.-L., Yu Q., Ming R. (2019). Comparative Genomics Revealed the Gene Evolution and Functional Divergence of Magnesium Transporter Families in Saccharum. BMC Genom..

[50] Cho E.K., Choi Y.J. (2009). A Nuclear-Localized HSP70 Confers Thermoprotective Activity and Drought-Stress Tolerance on Plants. Biotechnol. Lett..

[51] Kose S., Furuta M., Imamoto N. (2012). Hikeshi, a Nuclear Import Carrier for Hsp70s, Protects Cells from Heat Shock-Induced Nuclear Damage. Cell.

[52] Heidari P., Puresmaeli F., Vafaee Y., Ahmadizadeh M., Ensani M., Ahmadinia H. (2023). Comparative Analysis of Phospholipase D (PLD) Gene Family in Camelina Sativa and Brassica Napus and Its Responses in Camelina Seedlings under Salt Stress. Agronomy.

[53] Puresmaeli F., Heidari P., Lawson S. (2023). Insights into the Sulfate Transporter Gene Family and Its Expression Patterns in Durum Wheat Seedlings under Salinity. Genes.

[54] Jeffares D.C., Penkett C.J., Bähler J. (2008). Rapidly Regulated Genes Are Intron Poor. Trends Genet..

[55] Kumar D., Chattopadhyay S. (2018). Glutathione Modulates the Expression of Heat Shock Proteins via the Transcription Factors BZIP10 and MYB21 in Arabidopsis. J. Exp. Bot..

[56] Price B.D., Calderwood S.K. (1992). Heat-induced Transcription from RNA Polymerases II and III and HSF Binding Activity Are Co-ordinately Regulated by the Products of the Heat Shock Genes. J. Cell. Physiol..

[57] Duan Y.-H., Guo J., Ding K., Wang S.-J., Zhang H., Dai X.-W., Chen Y.-Y., Govers F., Huang L.-L., Kang Z.-S. (2011). Characterization of a Wheat HSP70 Gene and Its Expression in Response to Stripe Rust Infection and Abiotic Stresses. Mol. Biol. Rep..

[58] Yu X., Mo Z., Tang X., Gao T., Mao Y. (2021). Genome-Wide Analysis of HSP70 Gene Superfamily in *Pyropia yezoensis* (Bangiales, Rhodophyta): Identification, Characterization and Expression Profiles in Response to Dehydration Stress. BMC Plant Biol..

[59] Chen J., Gao T., Wan S., Zhang Y., Yang J., Yu Y., Wang W. (2018). Genome-Wide Identification, Classification and Expression Analysis of the HSP Gene Superfamily in Tea Plant (*Camellia sinensis*). Int. J. Mol. Sci..

[60] Sung D.Y., Guy C.L. (2003). Physiological and Molecular Assessment of Altered Expression of Hsc70-1 in Arabidopsis. Evidence for Pleiotropic Consequences. Plant Physiol..

[61] Ono K., Hibino T., Kohinata T., Suzuki S., Tanaka Y., Nakamura T., Takabe T. (2001). Overexpression of DnaK from a Halotolerant Cyanobacterium Aphanothece Halophytica Enhances the High-Temperatue Tolerance of Tobacco during Germination and Early Growth. Plant Sci..

[62] Leborgne-Castel N., Dooren E.P.W.M.J.-V., Crofts A.J., Denecke J. (1999). Overexpression of BiP in Tobacco Alleviates Endoplasmic Reticulum Stress. Plant Cell.

[63] Sugino M., Hibino T., Tanaka Y., Nii N., Takabe T. (1999). Overexpression of DnaK from a Halotolerant Cyanobacterium Aphanothece Halophytica Acquires Resistance to Salt Stress in Transgenic Tobacco Plants. Plant Sci..

[64] Chaudhary R., Baranwal V.K., Kumar R., Sircar D., Chauhan H. (2019). Genome-Wide Identification and Expression Analysis of Hsp70, Hsp90, and Hsp100 Heat Shock Protein Genes in Barley under Stress Conditions and Reproductive Development. Funct. Integr. Genom..

[65] Zhang J., Li J., Liu B., Zhang L., Chen J., Lu M. (2013). Genome-Wide Analysis of the Populus Hsp90 Gene Family Reveals Differential Expression Patterns, Localization, and Heat Stress Responses. BMC Genom..

[66] Liu X., Chen H., Li S., Wang L. (2022). Genome-Wide Identification of the Hsp70 Gene Family in Grape and Their Expression Profile during Abiotic Stress. Horticulturae.

[67] Qi Y., Xie Y., Ge M., Shen W., He Y., Zhang X., Qiao F., Xu X., Qiu Q.-S. (2024). Alkaline Tolerance in Plants: The AT1 Gene and Beyond. J. Plant Physiol..

[68] Rao Y., Peng T., Xue S. (2023). Mechanisms of Plant Saline-Alkaline Tolerance. J. Plant Physiol..

[69] Bolser D.M., Staines D.M., Perry E., Kersey P.J. (2017). Ensembl Plants: Integrating Tools for Visualizing, Mining, and Analyzing Plant Genomic Data. Plant Genomics Databases. Methods in Molecular Biology.

[70] Gasteiger E., Hoogland C., Gattiker A., Duvaud S., Wilkins M.R., Appel R.D., Bairoch A. (2005). Protein Identification and Analysis Tools on the ExPASy Server.

[71] Sievers F., Higgins D.G. (2014). Clustal Omega. Curr. Protoc. Bioinforma..

[72] Nguyen L.T., Schmidt H.A., Von Haeseler A., Minh B.Q. (2015). IQ-TREE: A Fast and Effective Stochastic Algorithm for Estimating Maximum-Likelihood Phylogenies. Mol. Biol. Evol..

[73] Letunic I., Bork P. (2021). Interactive Tree Of Life (ITOL) v5: An Online Tool for Phylogenetic Tree Display and Annotation. Nucleic Acids Res..

[74] Kumar S., Stecher G., Li M., Knyaz C., Tamura K. (2018). MEGA X: Molecular Evolutionary Genetics Analysis across Computing Platforms. Mol. Biol. Evol..

[75] Lescot M., Déhais P., Thijs G., Marchal K., Moreau Y., Van De Peer Y., Rouzé P., Rombauts S. (2002). PlantCARE, a Database of Plant Cis-Acting Regulatory Elements and a Portal to Tools for in Silico Analysis of Promoter Sequences. Nucleic Acids Res..

[76] Pertea M., Kim D., Pertea G.M., Leek J.T., Salzberg S.L. (2016). Transcript-Level Expression Analysis of RNA-Seq Experiments with HISAT, StringTie and Ballgown. Nat. Protoc..

[77] Anders S., Pyl P.T., Huber W. (2015). HTSeq-A Python Framework to Work with High-Throughput Sequencing Data. Bioinformatics.

[78] Tarazona S., García F., Ferrer A., Dopazo J., Conesa A. (2012). NOIseq: A RNA-Seq Differential Expression Method Robust for Sequencing Depth Biases. EMBnet J..

[79] Li G., Ma Q., Tang H., Paterson A.H., Xu Y. (2009). QUBIC: A Qualitative Biclustering Algorithm for Analyses of Gene Expression Data. Nucleic Acids Res..

[80] Cantalapiedra C.P., Hernández-Plaza A., Letunic I., Bork P., Huerta-Cepas J. (2021). EggNOG-Mapper v2: Functional Annotation, Orthology Assignments, and Domain Prediction at the Metagenomic Scale. Mol. Biol. Evol..

[81] Supek F., Bošnjak M., Škunca N., Šmuc T. (2011). REVIGO Summarizes and Visualizes Long Lists of Gene Ontology Terms. PLoS ONE.

[82] Smoot M.E., Ono K., Ruscheinski J., Wang P.-L., Ideker T. (2011). Cytoscape 2.8: New Features for Data Integration and Network Visualization. Bioinformatics.

[83] Du Z., Su H., Wang W., Ye L., Wei H., Peng Z., Anishchenko I., Baker D., Yang J. (2021). The TrRosetta Server for Fast and Accurate Protein Structure Prediction. Nat. Protoc..

[84] Trott O., Olson A.J. (2010). AutoDock Vina: Improving the Speed and Accuracy of Docking with a New Scoring Function, Efficient Optimization, and Multithreading. J. Comput. Chem..

[85] Adasme M.F., Linnemann K.L., Bolz S.N., Kaiser F., Salentin S., Haupt V.J., Schroeder M. (2021). PLIP 2021: Expanding the Scope of the Protein–Ligand Interaction Profiler to DNA and RNA. Nucleic Acids Res..

[86] Wallace A.C., Laskowski R.A., Thornton J.M. (1995). LIGPLOT: A Program to Generate Schematic Diagrams of Protein-Ligand Interactions. Protein Eng. Des. Sel..

[87] Livak K.J., Schmittgen T.D. (2001). Analysis of Relative Gene Expression Data Using Real-Time Quantitative PCR and the 2^−ΔΔCT^ Method. Methods.

